# Acute 1,3-Butanediol Co-Ingestion with Carbohydrate Does Not Improve Endurance Performance in Chronically Ketogenic Male Athletes

**DOI:** 10.3390/nu18142388

**Published:** 2026-07-22

**Authors:** Matthew Carpenter, James Brouner, Owen Spendiff

**Affiliations:** 1 School of Life Sciences, Pharmacy and Chemistry, Kingston University London, London KT1 1LQ, UK; matt.carpenter@northampton.ac.uk (M.C.); james.brouner@kingston.ac.uk (J.B.); 2 School of Sport and Public Health, University of Northampton, Northampton NN1 5PH, UK

**Keywords:** RER, fat oxidation, exogenous ketones, 1,3-butanediol, ketogenic, ketosis

## Abstract

**Background/Objectives:** Exogenous ketone supplementation has been proposed to enhance endurance performance by altering substrate metabolism and sparing muscle glycogen. Prolonged carbohydrate restriction may upregulate ketolytic capacity and could therefore modulate the response to exogenous ketone ingestion. This study investigated the impact of increasing circulating ketone bodies through exogenous ketone supplementation on endurance performance in athletes chronically adapted to a ketogenic diet. **Methods:** Participants (*n* = 9 recreationally active males, with ≥1 year of ketogenic adherence) visited the lab four times. The first visit determined their $\dot{V}$O_2_max. The second visit accustomed them to test procedures. In a counterbalanced design, visits three and four tested the consumption of a 60 g carbohydrate beverage (CHO), or a beverage comprising 60 g carbohydrate and 0.5 g/kg 1,3-butanediol (CHO + BD). This was followed by a 60 min set-intensity exercise bout, followed by a 16.1 km time trial. **Results:** βHB significantly increased in the CHO + BD group (rest: 0.72 ± 0.33 mmol/L; post-exercise: 1.69 ± 0.30 mmol/L) compared to CHO (rest: 0.80 ± 0.53; post-exercise: 0.55 ± 0.30) (*p* < 0.001). There was no significant difference in time trial performance between groups (1606 ± 138 s; 1620 ± 134 s; *p* > 0.05). During set intensity exercise, there was no significant main effect of supplementation on lactate concentration, substrate oxidation, oxygen consumption at a fixed workload, RPE or heart rate (*p* > 0.05). However, glucose was significantly lower at minutes 40 and 60 of set intensity exercise in CHO + BD compared to CHO. **Conclusions:** No significant effect of 1,3-butanediol ingestion on endurance performance was detected in this sample of nine chronically ketogenic male athletes. βHB significantly increased from resting levels; however, time trial performance did not differ between conditions. Given the small sample and limited statistical power, these preliminary findings indicate no detectable ergogenic effect under the present conditions rather than evidence of no effect, and larger studies are warranted.

## 1. Introduction

Once considered metabolism’s ‘ugly duckling’ due to its association with diabetes [1], research has illustrated the role of ketones as a metabolically efficient substrate for extrahepatic tissue such as skeletal muscle, the heart and the brain [2,3,4]. The combustion of 100 g of glucose yields approximately 8700 g of ATP, compared with 10,500 g from 100 g of d-β-hydroxybutyrate (βHB) [5]. Unlike fatty acids, whose greater ATP yield per molecule is offset by a higher oxygen cost, β-hydroxybutyrate is theorised to offer a more favourable ratio of ATP resynthesised per unit of oxygen, comparable to that of carbohydrate, owing to its effect on mitochondrial redox state and the free energy of ATP hydrolysis [3,6]. Elevated βHB increases the free energy of ATP hydrolysis by reducing the mitochondrial NAD^+^/NADH couple while oxidising the coenzyme Q couple, thereby widening the redox span across the electron transport chain [7]. It has therefore been hypothesised that elevating ketone oxidation would enhance exercise efficiency [3,8,9]. Previous researchers have suggested that exogenous ketone ingestion may enhance exercise efficiency [10]; however, research is mixed, with a prior meta-analysis reporting no improvements in exercise efficiency following exogenous ketone ingestion [11].

In addition to their role as a metabolic substrate, ketone bodies can also have modulating effects on carbohydrate, lipid, and protein metabolism [12], as well as decreasing exercising lactate [5]. Previous research has demonstrated increased intramuscular triglyceride utilisation in addition to reduced rates of glycolysis, which seminal research demonstrated to have a glycogen-sparing effect [5]. However, findings on whether ketones may spare muscle glycogen are mixed [13]. Ketones may also alter the glycaemic response to carbohydrate, with ketone ingestion reducing blood glucose [14] and suppressing the glycaemic response to carbohydrate ingestion compared to a taste-matched, non-isocaloric placebo when consumed 30 min prior to a 75 g glucose bolus, with an 11% reduction in blood glucose area under the curve throughout the 120 min oral glucose tolerance test [15]. Since carbohydrate is commonly consumed pre-exercise to maintain blood glucose, liver glucose and muscle glycogen, it is therefore of interest to combine exogenous ketone ingestion with carbohydrate to enhance ecological validity of research.

Despite the physiological rationale, most findings on the effect of pre-exercise exogenous ketone ingestion are equivocal [16], with meta-analyses and systematic reviews indicating no benefit of exogenous ketone ingestion on endurance performance [16,17]. Some research also suggests performance impairment, likely due to the role of exogenous ketones in suppressing glycolytic flux [18,19]. Lack of an ergogenic effect may also be due to low rates of ketone oxidation, with isotope tracer research reporting ketones to provide just 2.48–4.46% total energy expenditure during endurance exercise, with the highest relative ketone oxidation occurring at 25% peak power output [10]. The lack of ergogenic effect may also be due to the cohorts studied in previous research, as the metabolic status of individuals can have a marked impact on substrate metabolism [20]. One method of increasing the capacity to oxidise ketone bodies may be to follow a ketogenic diet [21]. Prolonged endogenous ketosis may represent a metabolic phenotype that increases susceptibility to the ergogenic effects of exogenous ketones, with Whitfield et al. [22] suggesting that exogenous ketones may improve performance in ketogenic individuals by attenuating the impairment in oxygen consumption that is demonstrated following ketogenic interventions [23].

Previous research has found no benefit to exogenous ketone ingestion following five days of a ketogenic diet [22]. However, whole-body metabolic adaptations are likely to require a longer time on a ketogenic diet [22], and short duration (<10 day) ketogenic interventions repeatedly demonstrate impaired performance [24,25,26]. This may confound short-duration studies examining the impact of exogenous ketosis on endurance performance following adherence to a ketogenic diet. Ketogenic individuals do not routinely consume carbohydrate; however, recent research by the present authors reported improved endurance performance in ketogenic individuals following carbohydrate ingestion [27]. To the authors’ knowledge, this is the first study to investigate the effect of exogenous ketone co-ingestion with carbohydrate on endurance performance in individuals with ≥1 year of ketogenic adherence, providing a direct test of whether chronic dietary adaptation modulates the ergogenic potential of exogenous ketones. Therefore, this study aimed to assess the impact of exogenous ketone ingestion on physiology and endurance performance in chronically ketogenic individuals. The primary aim of this study was to assess the effect of acute 1,3-butanediol co-ingestion with carbohydrate on 16.1 km cycling time trial performance in keto-adapted endurance athletes. Secondary aims were to examine the accompanying physiological and metabolic responses, including blood glucose, blood lactate, respiratory exchange ratio, substrate oxidation, oxygen uptake ($\dot{V}$O_2_) at a fixed workload, heart rate, and rating of perceived exertion. It was hypothesised that 1,3-butanediol co-ingestion would impact time trial performance relative to carbohydrate alone.

## 2. Materials and Methods

### 2.1. Experimental Approach

This study used a single-blind, counterbalanced, placebo-controlled crossover design to assess the acute effect of 1,3-butanediol co-ingestion with carbohydrate on endurance performance in chronically ketogenic male athletes. The primary outcome was 16.1 km time trial (TT) completion time. Secondary outcomes were physiological and metabolic responses during 60 min of steady-state exercise, including blood glucose, blood lactate, β-hydroxybutyrate (βHB), oxygen consumption ($\dot{V}$O_2_) at a fixed workload, respiratory exchange ratio (RER), substrate oxidation, heart rate (HR), and rating of perceived exertion (RPE). Each participant completed four laboratory visits: an incremental exercise test to determine maximum aerobic power output ($\dot{V}$O_2_max, visit 1), a familiarisation session replicating the experimental trial protocol (Visit 2), and two experimental trials in which either a carbohydrate (CHO) or a carbohydrate plus 1,3-butanediol (CHO + BD) beverage was consumed in a counterbalanced order (Visits 3 and 4). Experimental trials were separated by a 6-day washout. The 6-day washout period between trials was selected to allow participants to return to their habitual ketogenic state before the second trial.

This study was approved by the Kingston University research ethics committee (ethics code: 2678) and all procedures conformed to the Declaration of Helsinki. All participants received a written information sheet outlining the study procedures, risks, and benefits, and provided written informed consent prior to enrolment.

### 2.2. Participants

Nine recreationally active males (4× runners, 4× cyclists, 1× triathlete) volunteered to participate in the study. All participants were male; this reflected the volunteers eligible within the specialised keto-adapted endurance-athlete population during the recruitment period. Mass, lean mass and body fat percentage were measured using a bioelectrical impedance analysis device (Tanita MC-980MA PLUS BIA; Tanita Europe B. V. Amsterdam, The Netherlands) upon participants’ first visit to the laboratory, with participants only wearing light clothes following an overnight fast. Participants were informed of the study methodology before providing informed consent and completing health screening. To be eligible for the study, participants must have (1) adhered to a KD (defined as <50 g carbohydrate per day) for at least one year, (2) be free from illness, (3) body mass stable, (4) regularly complete a minimum of two endurance-based exercise sessions per week, (5) be >18 and (6) be a non-smoker and (7) have no history of eating disorders.

### 2.3. Dietary Adherence

To ensure adherence to a ketogenic diet, participants who volunteered were on self-described ketogenic diets and stated that daily carbohydrate consumption was <50 g/day, or <10% of total calories, in line with ketogenic diet definitions outlined by Kirkpatrick et al. [28]. To further ensure participants’ eligibility for the study, a three-day food diary was recorded using a mobile app (MyFitnessPal, Under Armour Inc., Baltimore, MD, USA) to ensure participants met the ketogenic diet definition. Participants’ dietary intake was cross-checked against the Kirkpatrick et al. [28] definition to ensure eligibility for the study. In addition, the resting capillary blood ketone value was taken upon the participants’ first visit to the lab using a finger prick ketone monitor (KetoMojo, 952 School Street 212, Napa, CA 94559, USA) to ensure a circulating ketone concentration of ≥0.3 mmol/L. This value was used based on previous research demonstrating a mean ketone concentration of 0.33–0.72 following a ketogenic intervention [29]. The Keto Mojo ketone metre has been demonstrated to be an accurate and reliable method of measuring blood ketones, demonstrating strong diagnostic performance in diagnosing ketosis [30].

Participants were instructed to not deviate from their current dietary pattern throughout the testing period. Baseline characteristics of the participants are outlined in Table 1.

### 2.4. Incremental Exercise Test

The incremental exercise test was performed at least 7 days before the first experimental trial. Testing was conducted in the fasted state to standardise pre-test conditions across all laboratory visits and to avoid the introduction of an acute carbohydrate feed that could confound substrate oxidation measurements in this keto-adapted cohort.

The incremental exercise test to volitional fatigue was conducted on a mechanically braked cycle ergometer (Monark model 894e, Monark Exercise AB, Vansbro, Sweden). Following a 5 min warm-up at 120 W, workload was increased by 30 W every minute until volitional exhaustion or until cadence fell below 60 rpm. Maximum power output (Wmax) was calculated using the equation of Kuipers et al. [31] as: last completed stage (W) + [time spent in the last incomplete stage (s)/60 (s)] × 30 (W). Expired gas was analysed continuously (Jaeger, VIASYS Healthcare GmbH, Höchberg, Germany) to determine $\dot{V}$O_2_max.

### 2.5. Experimental Trials

Following the $\dot{V}$O_2_max test and familiarisation, participants completed two experimental trials (CHO and CHO + BD) separated by a 6-day washout. Condition order was allocated using systematic counterbalancing rather than independent randomisation of each participant: the order for the first participant was determined using a computer-generated randomiser (www.randomizer.org, accessed on 15 May 2021), and subsequent participants were assigned in alternating order to balance the number of participants receiving each condition first. This approach was selected to guarantee balanced condition order across a small sample, in which simple randomisation carries a substantial risk of order imbalance. Participants attended the laboratory between 08:00 and 11:00 following an overnight (≥10 h) fast, at the same time of day for both trials. They were instructed to refrain from vigorous exercise for 48 h prior to each trial and to maintain their habitual ketogenic dietary pattern (<50 g carbohydrate per day) throughout the testing block. No dietary standardisation was imposed: participants were not provided with standardised meals, and food intake was not prescribed, weighed, or monitored during the testing block. Adherence was verified by self-reported habitual pattern and by confirmation of nutritional ketosis via resting capillary β-hydroxybutyrate on arrival at each trial. Beverages were prepared in identical containers and were matched for taste and volume by the addition of stevia and methylsulfonylmethane to the CHO condition (Section 2.5.1). Participants were blinded to condition allocation throughout. Owing to laboratory access restrictions in place at the time of testing, beverage preparation, trial administration, and delivery of verbal cues were all performed by the lead investigator, who was therefore necessarily aware of condition allocation. Full double-blinding was consequently not feasible, and the study was single-blind.

**Figure 1:**
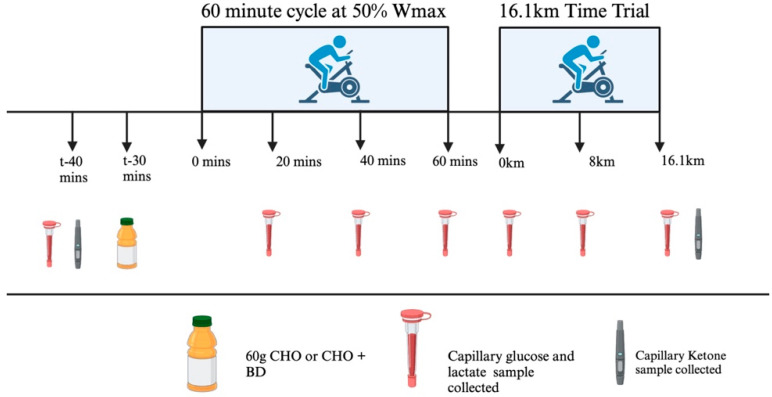
Schematic figure of experimental design. Created in BioRender. Carpenter, M. (2026). https://BioRender.com/f96s269.

The trial protocol is presented schematically in Figure 1 and comprised: (a) supplement ingestion, (b) 60 min of steady-state exercise, (c) a 15 min passive rest, and (d) a 16.1 km self-paced time trial.

#### 2.5.1. Supplementation

Thirty minutes before the start of steady-state exercise, participants consumed one of two isovolumetric beverages. In the CHO condition, the drink contained 60 g dextrose (100% glucose; MyProtein, The Hut Group, Cheshire, UK) prepared as an 8% solution with water, plus 1 g stevia extract (Bulk Powders, Essex, UK) and 3 g methylsulfonylmethane (MSM; Bulk Powders, Essex, UK) to match taste between conditions. In the CHO + BD condition, the drink contained the same components plus 0.5 g/kg body mass of 1,3-butanediol (Ketone-IQ™, HVMN, Miami, FL, USA), with water volume adjusted to maintain the 8% carbohydrate solution. The 60 g carbohydrate dose was selected in line with common pre-exercise carbohydrate recommendations (~1 g/kg body mass consumed 30–60 min pre-exercise) and to align with prior research investigating exogenous ketone co-ingestion with carbohydrate.

#### 2.5.2. Steady-State Exercise

Following the 30 min post-ingestion period, participants completed 60 min of continuous cycling on a cycle ergometer (Monark model 894e) at a fixed external workload of 50% Wmax (mean 142 ± 22 W). Participants cycled at a self-selected cadence between 70 and 90 rpm, monitored continuously via the Monark digital display; verbal cues were provided if cadence deviated by more than ±5 rpm from the target.

Capillary blood samples were taken at discrete time points (minutes 20, 40, and 60) and analysed using a Biosen C-Line Sport analyser (EKF Diagnostic Sales GmbH, Barleben, Germany) to determine lactate and glucose concentrations. Rating of perceived exertion (RPE) was recorded at each sampling time point using the 6–20 Borg scale [32], and heart rate was monitored continuously using a chest-strap monitor (Polar H7, Polar Electro Oy, Kempele, Finland). Capillary β-hydroxybutyrate was measured pre-exercise (upon arrival) and post-exercise (immediately after the time trial). Expired gases were collected continuously throughout the steady-state bout via an automatic gas analyser (see Section 2.5.4).

#### 2.5.3. Time Trial

Following a 15 min passive rest, participants completed a 16.1 km self-paced time trial on a Wattbike Pro (Wattbike Ltd., Nottingham, UK). During the time trial, participants received verbal feedback on distance covered at 4 km intervals only; elapsed time, power output, heart rate, and cadence were obscured to standardise pacing feedback across conditions. Capillary blood samples for lactate and glucose were taken at 0 km (start), 8 km, and immediately upon completion. Heart rate and RPE were recorded at 4 km, 8 km, and 16.1 km.

#### 2.5.4. Gas Exchange and Substrate Oxidation

Oxygen consumption and carbon dioxide exhalation were measured, with participants wearing a mask attached to an automatic gas analyser (VIASYS GmbH, Eric Jaeger, Hoechberg, Germany). These data were averaged using both absolute and relative data, with relative data calculated based on participants’ weight on arrival. Data were reported for minutes 0–20, 20–40 and 40–60 to analyse metabolic data between groups, with data analysed as mean values over each 20 min timepoint, as well as whether participants were in a metabolic steady state. These data were also measured during the TT and reported at ~4 km intervals (0–4 km, 4–8 km, 8–12 km, 12–16.1 km).

Substrate oxidation rates were calculated using the non-protein respiratory quotient, developed and updated by Peronnet and Massicotte [33], with the fat oxidation and carbohydrate oxidation calculated as follows:

$$\mathrm{The}\;\mathrm{oxidation}\;\mathrm{rate}\;\mathrm{of}\;\mathrm{carbohydrates}=4.585\;\times \;{\dot{V}CO}_{2}\;L\;\min^{-1}\;-\;3.226\;\times \;{\dot{V}O}_{2}\;L\;\min^{-1}$$

$$\mathrm{The}\;\mathrm{oxidation}\;\mathrm{rate}\;\mathrm{of}\;\mathrm{fats}=1.695\;\times \;{\dot{V}O}_{2}\;L\;\min^{-1}\;-\;1.701\;\times \;{\dot{V}CO}_{2}\;L\;\min^{-1}$$

These equations assume that protein oxidation and ketone body oxidation contribute negligibly to whole body gas exchange, and that other metabolic processes involved in the production and utilisation of oxygen and carbon dioxide are negligible relative to glucose and fatty acid oxidation. Ketone oxidation was not measured in the present study, and no adjustment was made for it. Consequently, these calculations may underestimate total substrate oxidation and misattribute the ketone-derived contribution, particularly in keto-adapted participants in whom ketolytic capacity is upregulated. The implications of these assumptions for interpretation of the reported oxidation rates are addressed in the Discussion.

### 2.6. Statistical Analysis

Owing to the specialised nature of the keto-adapted athlete population, nine participants were recruited, consistent with comparable mechanistic crossover investigations (n = 9–11) [34,35]. A sensitivity analysis (G*Power 3.1.9.7, Heinrich-Heine-Universität Düsseldorf, Düsseldorf, Germany) indicated that, with the achieved sample of nine participants, the study was powered (80%, α = 0.05) to detect only large within-participant effects (dz ≈ 1.07). This represents a retrospective sensitivity analysis rather than a prospective a priori sample size justification; the sample size was determined by the availability of eligible participants within this specialised population. Consequently, the study was not powered to detect small or moderate within-participant effects, and non-significant findings should be interpreted as an absence of detectable effect rather than evidence of no effect. The corresponding risk of type II error is addressed in the Discussion.

All data are expressed as mean ± standard deviation (SD). All statistical analyses were performed using IBM SPSS Statistics, version 30 (IBM Corp., Armonk, NY, USA) (IBM Corp., Armonk, NY, USA). Normality was assessed using the Shapiro–Wilk test; all variables met the assumption of normality (*p* > 0.05), and parametric analyses were therefore applied throughout. A paired sample T-test was used to test the effect of supplementation on time trial performance. In instances where time was a factor, a two-factorial repeated measures ANOVA was used to analyse the effect of supplementation and time, in addition to analysing the presence of any interaction. Mauchly’s test of sphericity was used to ensure homogeneity of variance, and in instances in which this test was violated, a Greenhouse-Geisser correction factor was used. Significance was set at *p* < 0.05. Post hoc Bonferroni correction was used when significant main effects or interactions were present to reduce the risk of type 1 error. *p*-values are reported alongside effect size calculations where appropriate. Effect size was measured using partial eta-squared (η^2^p). Confidence intervals for between-condition comparisons are presented as the CHO + BD condition minus the CHO condition. Values were interpreted using the conventions of Cohen [36]: small ≈ 0.01, medium ≈ 0.06, and large ≈ 0.14. For pairwise comparisons, Cohen’s d was interpreted as small (0.2), medium (0.5), and large (0.8).

## 3. Results

### 3.1. Blood Ketone Concentration

Blood ketones increased in the CHO + BD condition from resting levels (0.72 ± 0.33) to post-exercise (1.69 ± 0.30) (mean increase 0.97 mmol/L, 95% CI [0.72, 1.22], *p* < 0.001). There was no change in ketones in the carbohydrate group (rest: 0.80 ± 0.53; post-exercise: 0.55 ± 0.30). There was also an interaction between groups (*p* < 0.001).

### 3.2. Set Intensity Exercise

**Figure 2:**
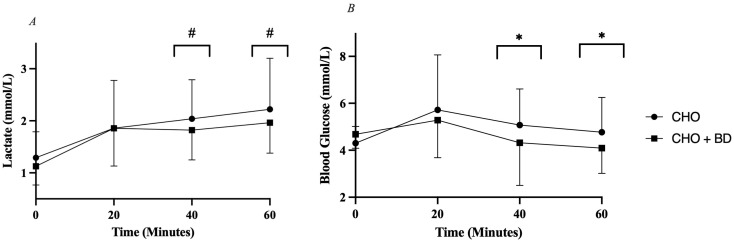
(**A**) Blood lactate across set-intensity exercise bout. # represents a main effect for time, with increased lactate compared to the 0 min timepoint. (**B**) Blood glucose across conditions throughout the set-intensity exercise bout. * represents a significant difference between supplemental groups at timepoints 40 and 60. Abbreviations: CHO = carbohydrate; CHO + BD = carbohydrate + 1,3-butanediol.

There was no significant main effect of supplementation on blood glucose concentration (*p* = 0.051; ηp^2^ = 0.39; CI [−0.74, 0.01]) or over time (*p* = 0.145; ηp^2^ = 0.23); however, there was a significant interaction between supplement and time factors (*p* = 0.023; ηp^2^ = 0.32). Blood glucose was significantly lower in the CHO + BD condition than the CHO condition at 40 min (95% CI [−1.29, −0.29], *p* = 0.005) and 60 min (95% CI [−1.17, −0.17], *p* = 0.014). There was no significant main effect of supplemental condition on lactate concentration (*p* = 0.500; ηp^2^ = 0.05, CI [−0.68, 0.36]). There was an effect of time on lactate concentrations (*p* < 0.001; ηp^2^ = 0.54). Lactate and glucose concentrations are outlined in Figure 2, below.

There was no significant main effect of time or supplementation on RER (time: *p* = 0.140; ηp^2^ = 0.43; supplement: *p* = 0.990; ηp^2^ < 0.01; 95% CI [−0.06, 0.06]), fat oxidation (time: *p* = 0.947; ηp^2^ < 0.01; supplement: *p* = 0.741; ηp^2^ = 0.01; 95% CI [−0.31, 0.23]) or carbohydrate oxidation (time: *p* = 0.269; ηp^2^ = 0.15; supplement: *p* = 0.895; ηp^2^ < 0.01; 95% CI [−0.59, 0.67]) throughout the set intensity exercise bout. Heart rate increased significantly over time (*p* = 0.010; ηp^2^ = 0.73); however, there was no significant main effect of supplementation (*p* = 0.512; ηp^2^ = 0.05; CI [−9.4, 5.1]). Relative $\dot{V}$O_2_, absolute $\dot{V}$O_2_, and %$\dot{V}$O_2_max all increased throughout the 60 min exercise bout (Relative $\dot{V}$O_2_: *p* < 0.01; ηp^2^ = 0.57, absolute $\dot{V}$O_2_: *p* = 0.030; ηp^2^ = 0.51, %$\dot{V}$O_2_max: *p* < 0.001; ηp^2^ = 0.60); however, there was no significant main effect of supplementation (Relative $\dot{V}$O_2_: *p* = 0.363; ηp^2^ = 0.10; CI [−3.45, 1.41]; absolute $\dot{V}$O_2_: *p* = 0.471; ηp^2^ = 0.06; CI [−0.21, 0.10]; %$\dot{V}$O_2_max: *p* = 0.456; ηp^2^ = 0.07; CI [−6.63, 3.26]). RPE increased over time (*p* = 0.003; ηp^2^ = 0.52), with no significant main effect of supplementation (*p* = 0.911; ηp^2^ < 0.01; CI [−0.78, 0.70]). These data are outlined in Table 2 below.

### 3.3. Time Trial

**Figure 3:**
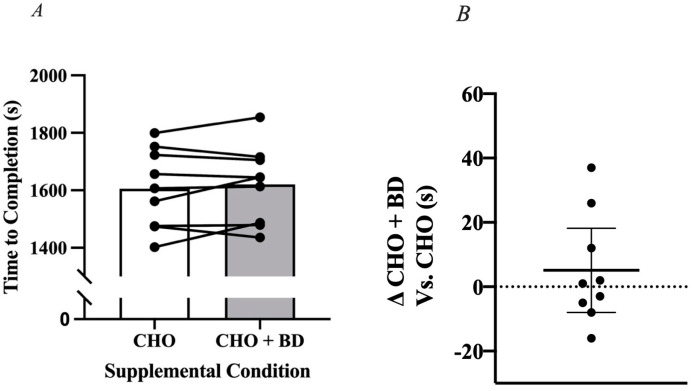
(**A**) Individual data points for time to completion during the 16.1 km time trial. Participants received CHO or CHO + 1,3-butanediol in a counterbalanced design. (**B**) Delta (change) in time to completion in a 16.1 km time trial. Points above the dotted line represent slower times in CHO + 1,3-butanediol. Data represent individual time to completion delta, in addition to delta mean ± standard deviation. Abbreviations: CHO = carbohydrate; CHO + BD = carbohydrate + 1,3-butanediol.

Supplemental condition had no significant effect on time to completion in the 16.1 km time trial (*p* = 0.40; CI [−22.8, 51.5], dz = 0.30) (CHO: 1606 ± 138 s; CHO + BD: 1620 ± 134 s) (Figure 3). There was no significant main effect of supplementation on mean power output (CHO: 196 ± 50 W; CHO + BD: 189 ± 42 W; 95% CI [−24.4, 9.7]) or peak power output (CHO: 423 ± 184 W; CHO + BD: 371 ± 169 W; 95% CI [−153.3, 48.2]).

Heart rate (HR) and ratings of perceived exertion (RPE) increased over time (HR: *p* < 0.001, ηp^2^ = 0.78; RPE: *p* < 0.001, ηp^2^ = 0.84), but with no significant main effect of supplementation (HR: *p* = 0.091, ηp^2^ = 0.31, 95% CI [−8.3, 2.3]; RPE: *p* = 0.712, ηp^2^ = 0.01, 95% CI [−0.7, 1.0]). Relative $\dot{V}$O_2_, absolute $\dot{V}$O_2_, % $\dot{V}$O_2_max and carbohydrate oxidation increased over time (Relative $\dot{V}$O_2_: *p* < 0.001, ηp^2^ = 0.60; absolute $\dot{V}$O_2_: *p* < 0.001, ηp^2^ = 0.59; %$\dot{V}$O_2_max: *p* < 0.001, ηp^2^ = 0.62; carbohydrate oxidation: *p* < 0.001, ηp^2^ = 0.55). There was no significant main effect of supplementation on Relative $\dot{V}$O_2_ (*p* = 0.139, ηp^2^ = 0.25; 95% CI [−7.76, 1.31]); absolute $\dot{V}$O_2_ (*p* = 0.143, ηp^2^ = 0.24; 95% CI [−0.47, 0.08]); %$\dot{V}$O_2_max (*p* = 0.141, ηp^2^ = 0.25; 95% CI [−13.94, 2.23]) or carbohydrate oxidation (*p* = 0.564, ηp^2^ = 0.04; 95% CI [−1.09, 0.64]). Fat oxidation decreased over time (*p* = 0.040; ηp^2^ = 0.37), with no significant main effect of supplementation (*p* = 0.907, ηp^2^ < 0.01; 95% CI [−0.21, 0.19]). RER increased over time (*p* < 0.001; ηp^2^ = 0.53), with no significant main effect of supplementation (*p* = 0.85; ηp^2^ < 0.01; 95% CI [−0.05, 0.04]). Metabolic and physiological data throughout the TT are outlined in Table 3 below.

**Figure 4:**
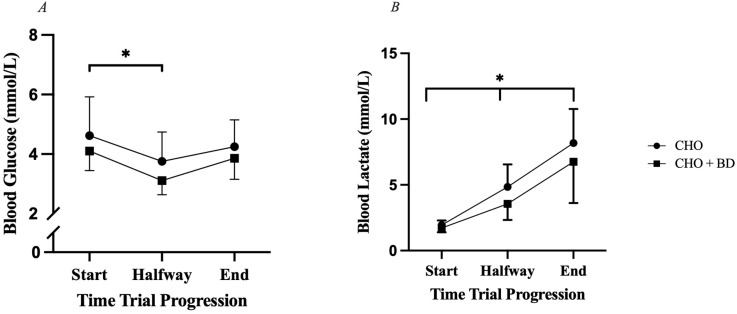
(**A**) Changes in blood glucose throughout the time trial. * represents a significant decrease in blood glucose between the start and 8 km measurements in the CHO + 1,3-butanediol group. (**B**) Blood lactate throughout the time trial. * represents a significant change over time at all timepoints. Data are mean ± SD. Abbreviations: CHO = carbohydrate; CHO + BD = carbohydrate + 1,3-butanediol.

Lactate concentration increased over time in both supplemental groups across all timepoints (*p* < 0.001; ηp^2^ = 0.87); however, there was no significant effect of supplement on lactate (*p* = 0.113; ηp^2^ = 0.28; 95% CI [−2.24, 0.29]). There was an effect of time on glucose concentration (*p* = 0.036; ηp^2^ = 0.33), with glucose lower at 8 km compared to 0 km in the CHO + BD group. There was no significant main effect of supplementation on glucose concentration (*p* = 0.065; ηp^2^ = 0.36; 95% CI [−1.07, 0.04]). Glucose and lactate concentrations from the time trial are outlined in Figure 4, below.

## 4. Discussion

This study detected no effect of 1,3-butanediol supplementation on 16.1 km time trial performance under the present conditions in chronically ketogenic individuals, despite a significant increase in blood ketone concentration following the CHO + 1,3-butanediol condition. This is the first study, to the authors’ knowledge, to assess the impact of exogenous ketone ingestion on performance in chronically ketogenic individuals. This finding supports previous research on ketone supplementation following carbohydrate restriction, showing no improvement in performance when taken pre-exercise [16], but adds the novel aspect of chronic adherence to a ketogenic diet. While chronic adherence to a ketogenic diet is often hypothesised to enhance the capacity for ketone body (KB) utilisation through upregulation of transporters and ketolytic enzymes, this has been demonstrated predominantly in rodent studies [37]. The present findings suggest that these metabolic adaptations do not necessarily translate into meaningful changes to oxygen consumption and performance. This absence of an ergogenic effect may be due to the low rates at which ketones are oxidised, particularly at high intensities, with ketone oxidation decreasing as exercise intensity increases [10]. Previous research indicates that even with prolonged ketogenic feeding, total ketone oxidation during exercise remains relatively low, contributing only a small fraction to overall energy expenditure [38].

Since ketone oxidation was not measured in the present study, any mechanistic account remains hypothetical. Low rates of ketone oxidation may nonetheless reflect only moderate expression of OXCT1 in skeletal muscle relative to the myocardium [39], and although ketogenic feeding has been reported to increase skeletal-muscle ketone utilisation in animal models, the overall effect is small [38]. Future research employing isotopic tracer methodology would allow the metabolic fate of ingested ketones to be determined directly.

We reported no significant main effect of supplementation on fat oxidation, carbohydrate oxidation or RER. These oxidation rates were estimated by indirect calorimetry under assumptions that may not fully hold in a keto-adapted cohort ingesting exogenous ketones (see Limitations). As circulating βHB was elevated only in the CHO + BD condition, any misattribution of ketone-derived carbon dioxide production would be expected to act more strongly in that condition, inflating apparent carbohydrate oxidation and deflating apparent fat oxidation relative to the CHO condition. This bias could therefore obscure a shift toward fat oxidation with 1,3-butanediol co-ingestion, and the possibility that a small true difference in substrate oxidation was not detected cannot be excluded. Although ketone bodies have been proposed to modulate glycolytic flux via signalling effects on the Randle cycle [40], such mechanisms were not directly assessed here, and their contribution to substrate oxidation in chronically keto-adapted individuals remains speculative. Future research should directly measure ketone oxidation rates alongside markers of intracellular signalling to understand any role in changes to carbohydrate and fat metabolism.

One further potential mechanism, though not directly assessed in the present study, is a disturbance to acid-base balance caused by exogenous ketone ingestion [41,42,43]. Ketone esters reduce blood pH owing to the acidity of βHB and acetoacetate [44], and a small but significant reduction has recently been reported for 1,3-butanediol in a clinical population [42], although this has not been observed for all ketone precursors [43]. On this basis, sodium bicarbonate has been co-ingested with ketone esters to offset this acidosis, with initial findings suggesting it may ‘unlock the ergogenic action of ketone monoesters’ [41], though subsequent work has been less supportive [18]. Blood pH was not measured here, and this evidence derives almost exclusively from ketone esters in non-keto-adapted individuals; whether acid-base disturbance contributed to the present findings, therefore, remains hypothetical. Future research incorporating direct measurement of blood pH alongside bicarbonate co-ingestion in keto-adapted individuals is warranted.

A further mechanism by which performance may be impacted is the change in glucose metabolism. This protocol involved ketogenic athletes consuming a moderate bolus (60 g) of carbohydrate, resulting in a novel physiological state in which carbohydrate is rapidly re-introduced in a cohort unaccustomed to carbohydrate ingestion, either with or without accompanying 1,3-butanediol. Previous research has suggested impaired glucose tolerance following carbohydrate restriction, possibly due to peripheral insulin resistance caused by reduced expression of glucose transporters [45], with this considered a ‘transient state’ [46] that is rapidly reversible upon carbohydrate re-introduction [47]. Exogenous ketones exert a blood glucose-lowering effect when taken prior to a meal [48], which may be a result of increased insulin secretion due to direct stimulatory action of βHB, reduced gluconeogenic precursors or reduced non-esterified fatty acid concentrations [48], although these mechanisms were not directly assessed here. This study supported the glucose-lowering effect of ketone ingestion, with a significant interaction effect, and blood glucose was significantly lower following CHO + BD compared to CHO alone (Figure 2B). This may be ergolytic, since hypoglycaemia has a negative impact on performance [49]. While the glucose-lowering effect may have clinical implications in type 2 diabetes [50,51], it does not present a potential ergogenic mechanism within endurance performance.

This suggests that 1,3-butanediol impacts the response to carbohydrate in ketogenic individuals, supporting the glucose-lowering effects of exogenous ketone ingestion. However, this did not translate to changes in substrate metabolism. We did not analyse the impact of glucose concentrations on time trial performance; however, reductions in blood glucose to hypoglycaemic levels impair endurance performance [49]; therefore, future work could examine pacing strategy by analysing the time-resolved power output profile across the time trial, to determine whether condition-related differences in substrate availability, such as reductions in circulating glucose following ketone ingestion, influence pacing and the distribution of effort during time trial performance.

No significant effect of 1,3-butanediol on lactate was detected during steady-state exercise or the time trial. This contrasts with research in ketone esters, which has demonstrated reductions in serum lactate concentration following ketone ingestion [5,52,53]. However, research in ketone precursors is mixed. Scott et al. [34] reported reduced lactate concentrations following 1,3-butanediol ingestion; however, subsequent research in endurance-trained cyclists completing 85 min of set intensity exercise revealed no change in lactate concentration [35]. No change in lactate was observed during continuous or time trial exercise; this may be attributed to the lower lactate concentrations observed in ketogenic athletes, likely due to reduced glycolytic activity [54]. As a result, a ketogenic cohort may be less susceptible to changes in lactate, particularly at moderate intensities. Prior research suggests ketones may also contribute to reduced lactate levels by decreasing glycolytic intermediates, such as pyruvate, in human skeletal muscle following ketone ingestion [4]. As these adaptations are also present following carbohydrate restriction, resulting in reduced glycolysis and a shift toward fat oxidation, even during high-intensity endurance [55,56], ketogenic athletes may not experience a further reduction in serum lactate with exogenous ketone ingestion.

One limitation of this study is the absence of direct measurement of ketone body oxidation. Substrate oxidation was estimated using indirect calorimetry equations that assume negligible protein and ketone oxidation, an assumption unlikely to hold fully in a keto-adapted cohort ingesting exogenous ketones, since though ketone oxidation remains low in ketogenic individuals, it is increased compared to keto-naïve individuals [38]. Because βHB oxidation yields a respiratory quotient higher than that of fat and approaching that of carbohydrate, any ketone-derived contribution to carbon dioxide production would be misattributed by these equations, inflating apparent carbohydrate oxidation and correspondingly deflating apparent fat oxidation. The likely magnitude of this bias is modest, as isotope tracer work indicates that exogenous ketone oxidation contributes approximately 2.5 to 4.5% of total energy expenditure during endurance exercise [10], which is small relative to the between-participant variability observed here. The absolute fat and carbohydrate oxidation rates reported should therefore be interpreted as estimates derived under these assumptions rather than as direct measurements. Future studies employing stable isotope tracer methodology would allow for direct quantification of ketone oxidation and allow a better understanding of the metabolic fate of ketone bodies. Another limitation is the absence of dietary standardisation. Participants followed their own self-selected ketogenic diets; no standardised meals were provided, and intake was not monitored during the testing block, with adherence verified via a three-day food diary completed before testing and confirmation of nutritional ketosis on arrival. Day-to-day variation in energy intake, macronutrient distribution, and pre-trial meal timing could therefore have influenced substrate availability and the metabolic responses observed and represents a risk of bias. Controlled feeding with standardised meals in the 24–48 h preceding each trial would substantially strengthen the internal validity of future work in this population. Consistent monitoring of nutrition throughout the study would enhance the reliability of the results. A further limitation is the use of a commercial app to monitor food intake, which may limit the validity of food intake data since nutrition apps may cause incorrect estimations due to their food composition databases [57]. Although a minimum endurance training frequency (≥2 sessions/week) was required for inclusion and aerobic fitness was characterised by $\dot{V}$O_2_max, detailed training-load data such as total weekly hours and volume were not recorded. Future studies capturing comprehensive training characteristics would allow finer contextualisation of inter-individual responses. A potential limitation concerns the ecological validity of the protocol. Participants arrived overnight fasted and consumed 60 g of carbohydrate (± 1,3BD) 30 min before exercise. This dose was selected to align with prior research on exogenous ketone co-ingestion with carbohydrate; however, it is lower than the carbohydrate intakes typically recommended for fuelling prolonged endurance exercise, and a single pre-exercise feed following an overnight fast differs from habitual race-day fuelling, which commonly includes carbohydrate intake during exercise. This approach was a methodological control to standardise pre-exercise metabolic state and isolate the effect of 1,3BD ingestion rather than to replicate competitive fuelling but may constrain ecological validity.

Recruitment was constrained by the specialised nature of the keto-adapted athlete population. Consequently, the small sample size (n = 9) limited statistical power and increased the risk of type II error. The observed effect on the primary outcome (dz = 0.30) was substantially smaller than the effect the study was powered to detect (dz ≈ 1.07), and smaller but physiologically meaningful effects may therefore not have been detected; non-significant results should be interpreted as inconclusive rather than as evidence of no effect. This study recruited male participants only. This is not solely a limitation of generalisability, as there are established sex differences in lipid oxidation, ketone body metabolism, and the hormonal response to exercise and to nutritional intervention. The present findings therefore apply to keto-adapted male athletes, and extrapolation to female athletes is not supported by these data. Research in keto-adapted female athletes, incorporating menstrual-cycle phase control, is required before any conclusions can be drawn for this population. Condition order was systematically counterbalanced rather than independently randomised for each participant. Whilst this guarantees balanced order in a small sample, the sequence was predictable after the first allocation and was not concealed from the lead investigator, who, owing to laboratory access restrictions at the time of testing, also prepared the beverages and administered the trials. Full double-blinding was therefore not feasible, and this represents a potential source of allocation and performance bias. A further consideration is the number of outcome variables analysed. Although Bonferroni correction was applied to post hoc comparisons following significant effects, no global correction was made across the full family of outcomes; secondary findings, including the observed glucose interaction, should therefore be regarded as exploratory and require confirmation in adequately powered work.

## 5. Conclusions

In this preliminary investigation of nine chronically ketogenic male endurance athletes (≥1 year ketogenic adherence), acute pre-exercise 1,3-butanediol co-ingestion produced no detectable improvement in 16.1 km time trial performance under the present conditions. Given the sample size and the associated power to detect only large effects, these data do not demonstrate the absence of an effect, and smaller but potentially meaningful differences cannot be excluded. Despite a clear rise in βHB concentrations following ingestion, oxygen consumption at a fixed workload was unchanged. A secondary observation was a significant interaction between time and supplement for blood glucose, with post hoc tests revealing lowering of blood glucose during steady-state exercise, consistent with the established glucoregulatory action of βHB, although the implications of this for performance in this population remain unclear. These findings should be interpreted as exploratory given the small, highly specific sample and the absence of direct measurement of ketone oxidation, which limits mechanistic interpretation. Future research employing larger samples and tracer-based quantification of ketone oxidation is warranted to determine whether long-term dietary adaptation influences the efficacy of exogenous ketones.

## Figures and Tables

**Table 1:** Characteristics of study participants.

	Participants (*n* = 9)
Age (years)	42 ± 9
Stature (cm)	176 ± 5
Body Mass (kg)	74.4 ± 6.1
Lean Mass (kg)	59.8 ± 4.7
Body Fat Percentage	12.8 ± 5.7
$\dot{V}$O_2_ max (mL/kg/min)	48.8 ± 7.5
% Calories from CHO	5 ± 3%
% Calories from Fat	68 ± 6%
% Calories from Protein	26 ± 5%
Resting Ketones (mmol/L)	0.8 ± 0.5
Time on self-reported Ketogenic Diet (months)	27 ± 17

**Table 2:** Metabolic and physiological data collected in a 60 min set-intensity exercise bout conducted at 50% Wmax.

	Condition	0–20 min	20–40 min	40–60 min
RER	CHO	0.83 ± 0.07	0.83 ± 0.06	0.83 ± 0.06
	CHO + BD	0.83 ± 0.02	0.84 ± 0.03	0.83 ± 0.02
$\dot{V}$O_2_ (mL/kg/min)	CHO	29.8 ± 4.8	30.9 ± 5.2 *	31.5 ± 6.0 *
	CHO + BD	28.9 ± 4.3	30.1 ± 4.3 *	30.1 ± 4.3 *
$\dot{V}$O_2_ (L/min)	CHO	2.23 ± 0.4	2.30 ± 0.4 *	2.29 ± 0.4
	CHO + BD	2.16 ± 0.3	2.25 ± 0.3 *	2.25 ± 0.3 *
%$\dot{V}$O_2_ max	CHO	61 ± 8%	63 ± 9% *	64 ± 9% *
	CHO + BD	60 ± 10%	62 ± 9% *	62 ± 9% *
Fat Oxidation (g/min)	CHO	0.62 ± 0.32	0.62 ± 0.33	0.62 ± 0.29
	CHO + BD	0.57 ± 0.17	0.58 ± 0.16	0.58 ± 0.18
CHO Oxidation (g/min)	CHO	1.32 ± 0.74	1.41 ± 0.77	1.40 ± 0.74
	CHO + BD	1.36 ± 0.49	1.45 ± 0.41	1.43 ± 0.48
Heart Rate (bpm)	CHO	136 ± 16	140 ± 16 **	144 ± 16 **
	CHO + BD	135 ± 14	138 ± 14 **	141 ± 16 **
RPE	CHO	10.1 ± 1.6	11.4 ± 2.1 **	12.8 ± 3.1 **
	CHO + BD	10.3 ± 1.7	11.4 ± 2.1 **	12.5 ± 3.6 **

Data are mean ± standard deviation. Significance was set at *p* < 0.05. * represents difference compared to 0–20 min. ** represents differences compared to all other time points. Abbreviations: RER = respiratory exchange ratio; RPE = rating of perceived exertion; βHB = β-hydroxybutyrate; BD = 1,3-butanediol; CHO = carbohydrate; $\dot{V}$O_2_ = oxygen consumption; HR = heart rate.

**Table 3:** Metabolic and physiological data collected during a 16.1 km self-paced cycle ergometer time trial.

	Condition	0–4 km	4–8 km	8–12 km	12–16.1 km
RER	CHO	0.84 ± 0.08	0.87 ± 0.06 *	0.89 ± 0.06	0.90 ± 0.06 *
	CHO + BD	0.84 ± 0.05	0.88 ± 0.06 *	0.88 ± 0.05 *	0.88 ± 0.05 *
$\dot{V}$O_2_ (mL/kg/min)	CHO	37.1 ± 7.8	39.4 ± 7.6 *	40.5 ± 5.9 *	41.1 ± 4.7 *
	CHO + BD	32.8 ± 6.5	36.9 ± 7.3 *	36.3 ± 8.1	39.2 ± 5.3 *
$\dot{V}$O_2_ (L/min)	CHO	2.8 ± 0.5	2.9 ± 0.4	3.0 ± 0.3 *	3.0 ± 0.3 *
	CHO + BD	2.4 ± 0.3	2.8 ± 0.5 *	2.8 ± 0.5 *	2.9 ± 0.4 *
%$\dot{V}$O_2_ max	CHO	76 ± 13	81 ± 13 *	84 ± 12 *	85 ± 12 *
	CHO + BD	69 ± 18	77 ± 17 *	76 ± 20	82 ± 15 *
Fat Ox. (g/min)	CHO	0.69 ± 0.33	0.54 ± 0.30 *	0.50 ± 0.32	0.47 ± 0.33 *
	CHO + BD	0.60 ± 0.19	0.49 ± 0.21 *	0.51 ± 0.22	0.56 ± 0.27
CHO Ox. (g/min)	CHO	1.85 ± 1.42	2.46 ± 1.33 *	2.67 ± 1.13 *	2.83 ± 1.08 *
	CHO + BD	1.65 ± 0.86	2.42 ± 1.25 *	2.39 ± 1.16 *	2.44 ± 0.97 *
Heart Rate (bpm)	CHO	156 ± 14	165 ± 10	170 ± 10	178 ± 10
	CHO + BD	151 ± 14	161 ± 13	164 ± 12	178 ± 13
RPE	CHO	13.4 ± 2.1	14.5 ± 1.9 *	16.5 ± 1.5 **	18.6 ± 0.5 **
	CHO + BD	13.6 ± 2.1	15.3 ± 1.8 *	16.4 ± 1.5 **	18.3 ± 0.8 **

Data are mean ± standard deviation. Significance was set at *p* < 0.05. * represents a difference compared to 0–4 km. ** represents a difference compared to 0–4 km and 4–8 km. Abbreviations: RER = respiratory exchange ratio; RPE = rating of perceived exertion; βHB = β-hydroxybutyrate; BD = 1,3-butanediol; CHO = carbohydrate; $\dot{V}$O_2_ = oxygen consumption; HR = heart rate.

## Data Availability

The original contributions presented in this study are included in the article. Further inquiries can be directed to the corresponding author.
